# Why We Need a Stronger Focus on Women’s Health in Clinical Psychology and Psychological Treatment

**DOI:** 10.32872/cpe.15683

**Published:** 2024-09-30

**Authors:** Cornelia Weise, Carola Hajek Gross

**Affiliations:** 1 Clinical Psychology and Behavioral Health Technology, Department of Psychology, Friedrich-Alexander-Universität Erlangen-Nürnberg, Erlangen, Germany; 2 Division of Clinical Psychology and Psychotherapy, Department of Psychology, Philipps-University of Marburg, Marburg, Germany

Despite advances in healthcare, women’s health issues—both physical and mental—remain underrepresented, underserved, and often misunderstood. The field of “gender medicine” highlights that many diseases manifest differently across various genders^1^1For the purposes of this paper, 'women' refers to individuals with a uterus. (Mauvais-Jarvis et al., 2020). Yet these differences are frequently neglected, leading to suboptimal care and increased health risks for women. A striking example is cardiovascular disease: women are significantly more likely to die from heart attacks than men, partly because their symptoms, such as nausea, fatigue, and back pain, are easily overlooked compared to the classic chest pain seen in men (Mousavi et al., 2023; van Oosterhout et al., 2020). Moreover, women are often underrepresented in medical clinical trials, and even when included, the unique impacts of therapies on women are often overlooked (Kalathoor et al., 2024; Vitale et al., 2017). This bias reflects a broader issue: medicine often treats men as the default, neglecting the specific needs of women. While clinical psychology exhibits a more balanced gender representation, the bias in medicine carries over, when psychological perspectives are applied to women's medical issues.

## Women Face Unique Health Challenges That Significantly Impact Their Mental Health

Several women-specific factors increase the risk of mental health disorders, with reproductive events, gender-based violence, socioeconomic factors, and hormonal fluctuations being prominent examples. Reproductive events such as miscarriage, infertility, or fear of childbirth often cause intense emotional distress, contributing to anxiety and depressive symptoms (e.g., Cuenca, 2023). Likewise, perinatal post-traumatic stress disorder is often overlooked or inadequately managed despite evidence that trauma-informed maternity care can drastically improve outcomes for affected women (Horsch et al., 2024). Gender-based violence, including sexual assault and domestic abuse, have a profound impact on mental health and often lead to trauma-related disorders (e.g., Sediri et al., 2020). Socioeconomic challenges, such as the significantly higher poverty risk among single mothers (Hübgen, 2020; Lu et al., 2020) contribute to chronic stress and burnout, further elevating the risk of anxiety and depression (Soares et al., 2007). Fluctuations in hormonal balance, such as those occurring during the menstrual cycle, pregnancy, or menopause, can exacerbate or trigger psychological complaints (Albert & Newhouse, 2019; Behrman & Crockett, 2023; Nolan & Hughes, 2022). Premenstrual dysphoric disorder (PMDD) is one example where hormonal changes during the menstrual cycle can lead to severe depressive and anxiety symptoms. Hormonal fluctuations also affect stress-response systems, including cortisol and alpha-amylase levels (Hantsoo et al., 2023; Helpman, 2023) and are closely linked to other conditions such as endometriosis or polycystic ovary syndrome (PCOS). Those affected experience both debilitating physical symptoms (e.g. chronic pain), and significant emotional distress (Chen et al., 2021; Dutkiewicz et al., 2024; Silva et al., 2024). This dual burden often restricts participation in social, family and working life, exacerbating psychological distress and potentially resulting in financial strain, particularly for women juggling caregiving responsibilities (Della Corte et al., 2020). The COVID-19 pandemic further intensified these burdens, as many women faced disrupted routines, increased caregiving responsibilities, and limited access to healthcare services (Di Blasi et al., 2021). Despite their prevalence and substantial economic burden, these conditions often remain underdiagnosed or untreated (Azziz et al., 2005; Ruszała et al., 2022).

## A Critical Need for Gender-Specific Treatment Approaches

Women’s mental and physical health are deeply interconnected, necessitating treatment strategies based on a biopsychosocial understanding of their unique needs (Engert et al., 2020). Recent research indicates that psychotherapeutic interventions can alter biological markers, underscoring the potential for gender-sensitive approaches to enhance outcomes for female patients (Laufer et al., 2018). By integrating both psychological and physiological factors into treatment, we can more effectively address the full scope of women’s health conditions. For instance, our recent intervention for endometriosis, rooted in cognitive-behavioral therapy (CBT) and a biopsychosocial framework, has been well-received by patients, who appreciate the focus on psychological distress associated with the chronic condition (Schubert et al., 2022).

## Barriers to Effective Psychotherapeutic Support

Despite the clear need for gender-sensitive mental health care, several barriers prevent women from receiving adequate support. This is often due to a lack of awareness of the psychological impacts of their physical symptoms, or the fear that their distress will be dismissed (Salk et al., 2017). Compounding this issue, many healthcare providers are not adequately trained to identify and address gender-specific health concerns, creating significant gaps in care. Endometriosis provides a striking example of this problem: Symptoms are frequently trivialized with menstrual pain often dismissed as "normal". This misconception contributes to an alarming delay in diagnosis – an average of 10 years in German-speaking countries (Hudelist et al., 2012). Such delays are particularly concerning because prolonged unmanaged pain can lead to central sensitization, where the nervous system becomes hypersensitive to pain stimuli, making treatment more complex and less effective (Hudelist et al., 2012; Mechsner, 2022). Failure to address the psychological aspects of chronic conditions like endometriosis represents a missed opportunity to alleviate suffering and modulate the pain experience. While some promising multidisciplinary programs have been developed (e.g.; Cunningham et al., 2024; Weise et al., 2019), they remain underutilized.

## Towards a Gender-Sensitive Approach in Clinical Psychology

To improve women's health outcomes, clinical psychology must broaden its scope and address the unique mental health needs of women, particularly in relation to physical health conditions. This calls for stronger interdisciplinary cooperation between psychotherapists and medical practitioners, such as gynecologists and endocrinologists (Nagel et al., 2013). Medical professionals need training in gender-specific health to identify early signs of mental distress and facilitate referrals. Psychotherapists should develop expertise in conditions like endometriosis and PMDD, as well as in understanding the impact of hormonal changes across life stages on mental health.

Research suggests that synchronizing psychological interventions with menstrual cycle phases could enhance their effectiveness, given the influence of estradiol and progesterone (Nillni et al., 2021). However, the limited number of studies highlight the need for further research. Additionally, incorporating psychophysiological assessments (e.g., cortisol and oxytocin levels) could help refine psychotherapeutic approaches to better align with women’s stress-responses (Fischer & Zilcha-Mano, 2022).

Evidence-based treatments such as cognitive-behavioral therapy (CBT) have proven effective in reducing the psychological burden of chronic physical illnesses (e.g., Chalder et al., 2023; Weise et al., 2016). However, these interventions are still scarce in the field of women’s health and not widely accessible, particularly within resource-constrained public health systems (Hansen et al., 2023). Expanding such integrated interventions will improve care by ensuring more holistic and personalized treatment.

Despite the potential of integrated care models, particularly in the field of behavioral medicine, many women face significant barriers to timely mental health care. Time constraints, caregiving duties, and the stigma surrounding mental health issues often prevent women from seeking support. Addressing these barriers requires more flexibility in clinical psychology, such as offering flexible schedules, remote psychotherapy, individualized interventions based on the biopsychosocial model, or improved access to online interventions. Policymakers must also foster research in gender-specific health care to ensure treatments that address the needs of diverse genders. With growing awareness and emerging innovative approaches (e.g.; Cunningham et al., 2024; Weise et al., 2019), there is significant potential to improve women’s mental health.
